# Policy analysis of the protection of Iranian households against catastrophic health expenditures: a qualitative analysis

**DOI:** 10.1186/s12913-023-09275-0

**Published:** 2023-05-05

**Authors:** Maryam Hedayati, Iravan Masoudi Asl, Mohammadreza Maleki, Ali Akbar Fazaeli, Salime Goharinezhad

**Affiliations:** 1 Department of Health Services Management, School of Health Management and Information Sciences, Iran University of Medical Sciences, Tehran, Iran; 2 Department of Health Management, Policy and Economics, Tehran university of medical sciences, Tehran, Iran; 3 Preventive Medicine and Public Health Research Center, Psychosocial Research Institute, Iran University of Medical Sciences, Tehran, Iran

**Keywords:** Catastrophic health expenditures, Impoverishment, Policy analysis, Health policy triangle, Policy impact determinants, Iran

## Abstract

**Background:**

Despite the adoption of various policies and strategies in recent decades, the Iranian health system has not succeeded in protecting households against catastrophic health expenditures (CHE) and impoverishment. Accordingly, this qualitative study aimed to critically analyze current policies for reducing CHE.

**Methods:**

This qualitative study was conducted as a retrospective policy analysis based on a document review and semi-structured interviews with key informants between July to October 2022. Two theoretical frameworks were used, including the Analysis of Determinants of Policy Impact (ADEPT) model and Walt and Gilson’s “Policy Triangle framework.” The country's related documents were searched through databases. In total, 35 participants were interviewed. Interviews and documents were analyzed using directed content analysis in MAXQDA v12 software. Interobserver reliability, peer check, and member check were done to confirm the trustworthiness of the data.

**Results:**

Twelve main themes and 42 sub-themes emerged from the data. The findings revealed that policy accessibility, policy background, and a clear statement of goals influenced the policy process. However, resources, monitoring and evaluation, opportunities, and obligations negatively affected the implementation process. In addition, a policy analysis based on the policy triangle framework demonstrated that the main factors affecting the policy on reducing CHE in Iran were “conflicts of interest,” “contextual factors,” “monitoring and evaluation,” and “intersectoral relationship” factors.

**Conclusion:**

The present study reflected the multifaceted nature of the barriers to reducing CHE in Iran. The implementation of the policy on reducing CHE requires the political will to improve intersectoral collaboration, strengthen the stewardship role of the Ministry of Health, design monitoring and evaluation mechanisms, and prevent personal and organizational conflicts of interest.

**Supplementary Information:**

The online version contains supplementary material available at 10.1186/s12913-023-09275-0.

## Introduction

Universal health coverage (UHC) aims to provide quality essential health services and affordable essential medicines and vaccines for all individuals without experiencing undue financial hardship [1]. One of the critical components of UHC, an agreed target of the United Nations (UN) Sustainable Development Goal (SDG) 3.8 on health and well-being, is to achieve financial risk protection (FRP) by 2030 [2]. The main indicators of FRP are, the incidence of catastrophic health expenditure (CHE), mean positive catastrophic overshoot, the incidence of impoverishment, and the increase in the depth of poverty occurring due to high out-of-pocket (OOP) healthcare expenditure [3]. Indeed, CHE and the impoverishment effect of OOP are the outcomes of poor financing mechanisms [4].

Financial health protection has been defined by the World Health Organization (WHO) as the state wherein “direct payments to obtain health services should not expose people to financial hardship and should not threaten their living standards” [5]. According to the WHO, excessive dependency on direct OOP, access to resources, and inefficient and unfair use of resources are the most important problems to achieve UHC. So, adequate public healthcare financing is essential for countries to achieve UHC [6].

Households can be protected from CHE by the design of appropriate policy solutions to reduce a health system's reliance on OOP payments and create more financial risk protection [7]. It means that high healthcare costs might not be catastrophic if a household does not bear the total cost because the service is provided for free, at a subsidized price, or is covered by third-party insurance. On the other hand, even small healthcare costs can be financially disastrous for low income group with no insurance coverage [8]. Therefore, planning and policy-making seem essential for reducing CHE and the resulting inequality and also promoting access to health services, especially the timely provision of services and organizational access [9].

Protecting people from CHEs is widely accepted as a desirable objective of Iran's health policies. Iran adopted UHC as a priority for its health system to increase financial protection and equity in access to health care for its citizens. Since 2005, Iran has implemented a series of reforms aimed at achieving UHC. The last reform was Iran’s Health Transformation Program (HTP) in 2014. Despite the fact that equity in health financing and utilization has been emphasized in national plans, upper-level documents, and reforms in Iran and is regarded as a social preference, Iran's health financing relied heavily on people's own pockets [10]. Also, inefficient policies regarding financing and the current international economic sanctions and financial crisis in Iran are associated with a higher probability increase OOP payments and exposed households to CHEs in Iran [11]. Moreover, OOP payments affect the poorest households disproportionately, thereby exacerbating inequality [12].

The WHO recommends that OOP payments for health care should not make up more than 20% of total health expenditure (THE) To reduce the prevalence of CHE in a country [7]. OOP spending is the major payment strategy for healthcare in most low-and-middle-income countries, such as Iran. The OOP expenditure as a percentage of total health expenditure in Iran in 2019 was 37%. The OOP expenditure was 34% in medical services, 40% in rehabilitation services, 41% in long-term nursing services, 47% in ancillary services of medical care, and 68% in all kinds of medicines and other medical goods distributed to outpatients [13]. According to the core indicators and indicators on the health-related SDGs reported by the WHO, the population with CHE was 2.1% in 2019 and 3.7% in 2020, and the population impoverished due to OOP health expenditure was 0.5 in 2019 and 0.86 in 2020 [14]. Therefore, CHEs and OOP remain as a significant health financing challenge in Iran, and targeted policies to reduce CHEs have not been completely successful.

This study aimed to critically analyze the policies that intend to reduce households expose to CHEs in Iran. A set of predefined criteria was used in retrospective policy document analysis, which may also be beneficial making future CHE reduction policies. The CHE reduction policy was evaluated for alignment between policy statements and intended outcomes. To achieve study objectives, the Analysis of Determinants of Policy Impacts (ADEPT) [15] and the Walt & Gilson’s policy analysis triangle [16] models were used.

## Methods

### Study setting

The present study was conducted in the Islamic Republic of Iran a member of the WHO Eastern Mediterranean region (EMR). Iran is a lower-middle-income country, according to the World Bank's classification, with gross national income (GNI) per capita (current US$) of $ 4,010 in 2019 and $3,530 in 2021 and -2.7% GDP growth in 2019 and 4.7% in 2021 [17]. Over the past decades, the increase of OOP, CHEs and impoverishment have become major concerns for health policymakers in Iran. Therefore, Iran’s government and parliament have developed several policies to improve fair financing and reduce OOPs during the last two decades (e.g. Principles 29 and 43 of the Constitution, Universal Health Insurance Law (1994), the establishment of a board of trustees in educational hospitals (2013), the establishment of family physician and rural insurance programs (2012), full-time geographic programs for physicians (2010), bed insurance (2008), free treatment of vehicle accident victims (2008),, and Iran’s Health Transformation Program (HTP) (2014) are examples of such protecting policies. To reach the goals of HTP eight packages were developed [18]. Also, the 4^th^ National Development Plans (NDP) (2005–2009) [19] and 5^th^ NDP (2011–2015) [20], have highlighted the reduction of OOP proportion of THE from 50 to 30%, and the reduction of households’ exposure to CHEs to 1%. These targets in 6^th^ NDP (2017–2021) were justified from 58 to 25% and from 6 to 1% for OOPs proportion of THE and households’ exposure to CHE, to protect households against the financial burden of diseases [21]. Nevertheless, no significant improvement is made in practice, the OOP is still more than 25% and the prevalence of CHEs among Iranian households is still significantly higher than 1% and healthcare expenditures lead to the health impoverishment of a high percentage of Iranian households [22].

### Study design

This qualitative study conducted in 2022 to evaluate the policy options available to reduce the incidence of CHEs and impoverishment in Iran. Our research team collected information in two ways: (1) a formal review of related documents, press releases, and technical reports on public websites; and (2) a qualitative in-depth interviews with the key informants who could influence the direction and outcome of the polices [23, 24].

### Document review

Document review is a systematic procedure for reviewing or evaluating documents, which can be used to collect more data on the policies, identify relevant categories of analysis for the overall set of documents, interpret the body of documents, and explore the role of evidence in the policies [25]. This stage is a document review of national policy documents and articles relating to the policies on reducing CHEs in Iran. In order to identify all national policy documents related to reducing CHEs in Iran and to ensure the “confirmability” of the method two search strategies were, with no time limit, used [15].

First, we searched Google Scholar and Open Grey as well as key journals (International Journal of Health Policy and Management, Journal of Community Health Research, Journal of Health Administration, Journal of Evidence-Based Health Policy, Management & Economics, etc.) to detect policy documents and the relevant grey literature. Second, the official websites of the relevant organizations and stakeholders, including the Ministry of Health and Medical Education (MoHME), the Islamic Parliament Research Center (IPRC), the Planning and Budget Organization, the Social Security Organization (SSO), the Iran Health Insurance Organization (IHIO), private supplementary insurance companies, the Ministry of Cooperatives, Labor, and Social Welfare, the Social Security Office of the Armed Forces, the Foundation of Martyrs and Veterans Affairs, the government, and the Eastern Mediterranean Region Office (EMRO), were appraised to explore relevant national documents, laws, framework, agreement, implementation plan, strategic plan, operational plan, action plans, strategy, roadmap, strategic priorities document, policy, program guidelines, and program plan.

All potentially relevant information was downloaded for analysis. Search terms included “OOP”, “out-of-pocket”, “catastrophic health expenditure”, “CHE”, “impoverishment”, “policy development”, “policy implementation”, and “health policy”. Each of these keywords was used separately in the Persian language. We searched PubMed and SCOPUS with the abovementioned keywords in combination with “Iran” AND “policy OR program,” in order to find any related articles. In parallel with the web-based search, a stakeholder-based search strategy was used, which is suggested by Jeanfreau et al. (2010) as a way to increase the credibility and trustworthiness of qualitative research [26]. All identified policy stakeholders were contacted telephonically and by email by the first author (MH) to obtain the most recent policy-related documents. Finally, we reviewed the references of each collected document to identify other potential documents to include.

Each document was then reviewed and summarized in a data collection sheet that included the title of the document, the date, the actors, whether the evidence was used or not, and a summary. The review of documents was conducted independently by two different reviewers from the research team (MH and I MA) to achieve consensus. Thematic analysis was used to analyze the collected data. Codebooks were developed and themes and sub-themes that emerged from the data were repeatedly reviewed using research questions. In this study, Braun and Clarke’s methodology was adopted, which consisted of six phases (namely getting familiar with data, establishing initial codes, discovering, evaluating, labeling emerged themes, and preparing a final report) [27]. Document reviews and thematic analyses were held during July and August 2022.

### Key informant interviews

To increase the credibility and trustworthiness of qualitative research, we supplemented the document review with conducted semi-structured interviews with stakeholders and key informants [26]. Interviews and thematic analyses were held during September and October 2022. All interviews were held face-to-face and via telephone, each lasting between 30 and 45 min. All participants were interviewed only once. All interviews were performed in a quiet room without any third parties by the first author (MH). Prior to the interviews, the participants received a thematic interview guide; this was a conscious decision of the research team, as the more-or-less prepared answers ensured completeness. The objectives of the research, as well as the reasons for and interests in the research topic, were first described to the participants. The interview guide consisted of a series of open-ended questions created for this study by the researchers. The interview guide was subsequently based on an adapted version of the ADEPT model [15] and Walt and Gilson’s policy analysis triangle framework [16] (see Supplementary 1). The interview guide was tested in a pilot interview for comprehensibility. The participants’ perceptions of the interviewer, including her professional role, can influence the interaction, and hence the information that is revealed [28]. So, the participants were informed about the researcher’s professional background as a PhD student in health policy at the time of the study. Recruitment was considered complete when saturation was achieved. Data saturation means no new information or themes are discovered in the empirical data, indicating that the data collection may finish [29].

### Participants

We intended to interview those who played a substantial role in the initiation and drafting of the policies on reducing CHE. Predefined selection criteria required the interviewees to be civil servants or representatives from universities, researchers, academics, and policymakers at the micro, meso, and macro levels at the MoHME, IHIO, Iran Medical Council, Parliament, Armed Forces Insurance Organization, SSO, and Academy of Medical Sciences, and to be substantially involved in the development process of the Iran CHE reduction strategy. The list of participants was prepared by reviewing relevant documents and was updated by interviewees using the purposive sampling and snowballing method. To confirmability, the member review was done by co-authors and participants. Because of the familiarity of the research team members with the health economic and health policy field and also the health financing system in Iran, they had enough knowledge about the potential participants.

A total of 35 participants were identified to explore the opinions and perceptions of stakeholders (see supplementary 2). None of the interviewed participants identified additional actors that would have been relevant to our study. The participants were contacted via email and an instant messaging application, and the date, time, and method of the interviews were determined. Before each interview, written informed consent forms were signed after explaining the objectives of the study and providing general information about the interviewer. The forms were then collected from the participants. Furthermore, individuals were guaranteed to be anonymous throughout the research, and they were free to leave the study at any stage if they wished.

### Conceptual framework of the study

Two complementary theoretical frameworks were used to inform data collection and analysis in this study: the ADEPT (Analysis of Determinants of Policy Impact) model [15] and the policy analysis triangle [16]. The ADEPT framework provides a framework for describing components of the CHE reduction policy that are expected to increase the policy’s potential impact on intended outcomes. While the Policy Triangle analyzes the content of policies, the range of actors involved, the contextual factors, and the policy processes related to reducing CHE. These two frameworks worked together to provide a comprehensive analysis of health financing and the specific policies aimed at reducing CHE.

Rutten et al.’s (2011), in developing the ADEPT model, have adapted this theory to explain the impact of a policy based on four determinants (Goals, Obligations, Resources, and Opportunities) Rutten and colleagues (2011), in developing the ADEPT model, have adapted this theory to explain the impact of a policy based on four determinants (Goals, Obligations, Resources, and Opportunities). This model was used to evaluate the CHE reduction policy for alignment between policy statements and intended outcomes. Also by using these criteria we analyzed all obtained policy documents to explain the policy's successes or failures. The ADEPT model aims to explain and influence policy development and policy impact implementation [15]. Rutten et al.’s, describe the impact of a policy as consisting of policy output (the actions taken by the government once the policy is adopted) and policy outcomes (the health effects on the population level once an action is taken).This predefined set of criteria was chosen because it has been validated as a useful tool in analyzing health policy [15].

Cheung et al. in a health policy analysis study added and validated three new criteria to those of Rütten et al. after a literature review and consultation with experts in the field [30]. The following is an explanation of amendments were made to the criteria of Rütten et al.’s:

- Accessibility: Document accessibility may be a facilitator or barrier to the usefulness and implementation of policy [30].
- Policy background: Policy background encompasses the consideration of scientific results, so the Rütten criterion ‘scientific results demand the action’ has been incorporated into this section. Sources may be of different types: authority (e.g. persons, books, articles), quantitative or qualitative analysis, and deduction (premises established from an authority, observation, intuition, or all three) [30].
- Monitoring and evaluation: Independent evaluation strengthens the analyses’ credibility. Data collection before and after implementation also increases the credibility of the evaluation [30].

**Fig. 1:**
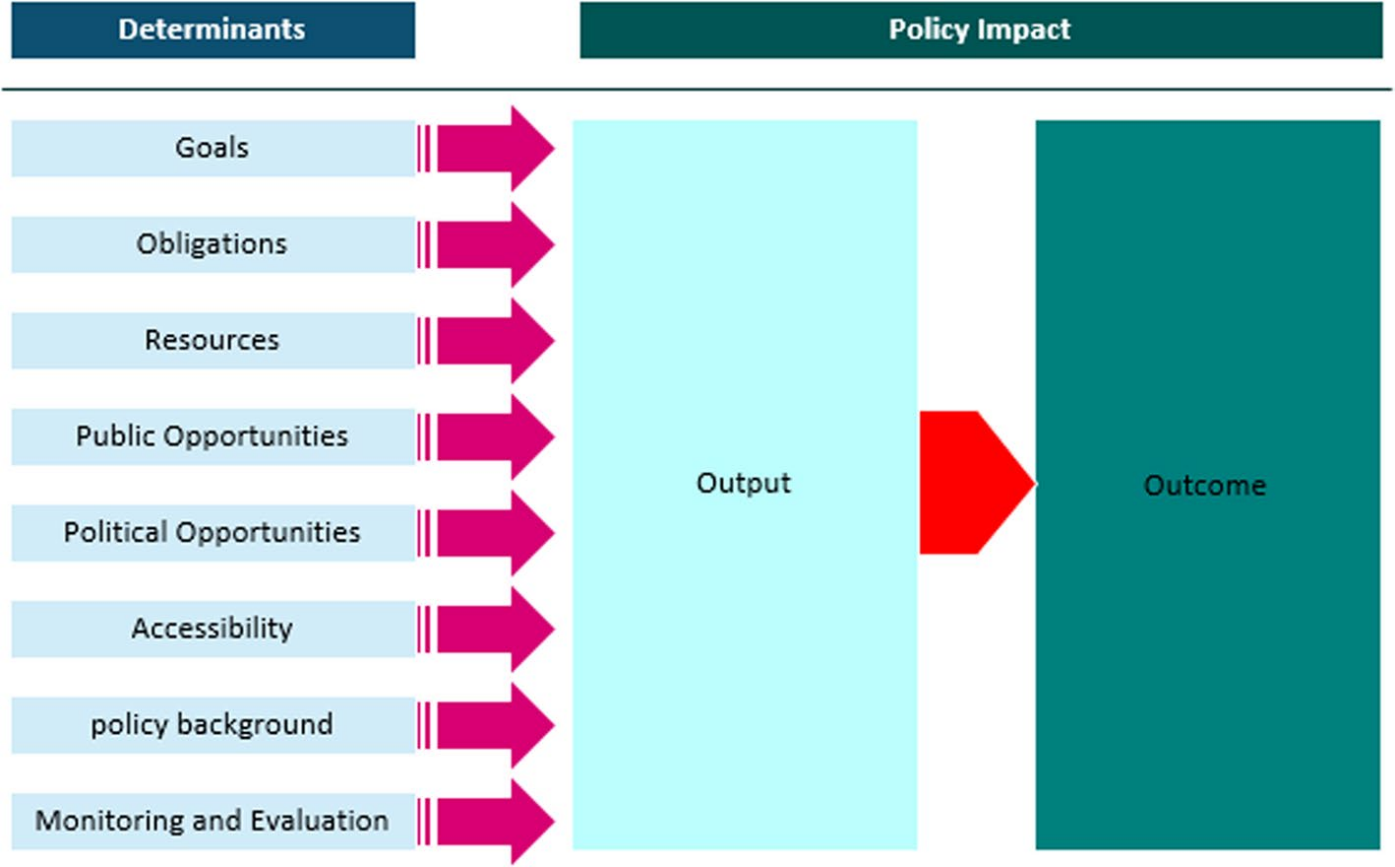
The ADEPT model. Source: Cheung et al. (2010) [30]

Following the adapted ADEPT model that Cheung et al. had used in the health policy document review [30], we also consider three additional determinants: accessibility, policy background, and monitoring and evaluation to evaluate policy determinants and outcomes (see Fig. 1). These criteria, taken together, help to gain an understanding of the specific policy situation for that determinant. These criteria, help to gain an understanding of the specific policy situation for that determinant, enable policymakers to review how closely policy intentions are reflected in their documents, and reveal how policy documents may be amended to take into account lessons learned from successes and failures experienced [30] (see Supplementary 3). Furthermore, the ADEPT model draws inspiration from Kingdon’s multiple streams approach [31], Baumgartner and Jones’ punctuated equilibrium framework [32], and Kiser and Ostrom’s institutional analysis and development framework [33].

**Fig. 2:**
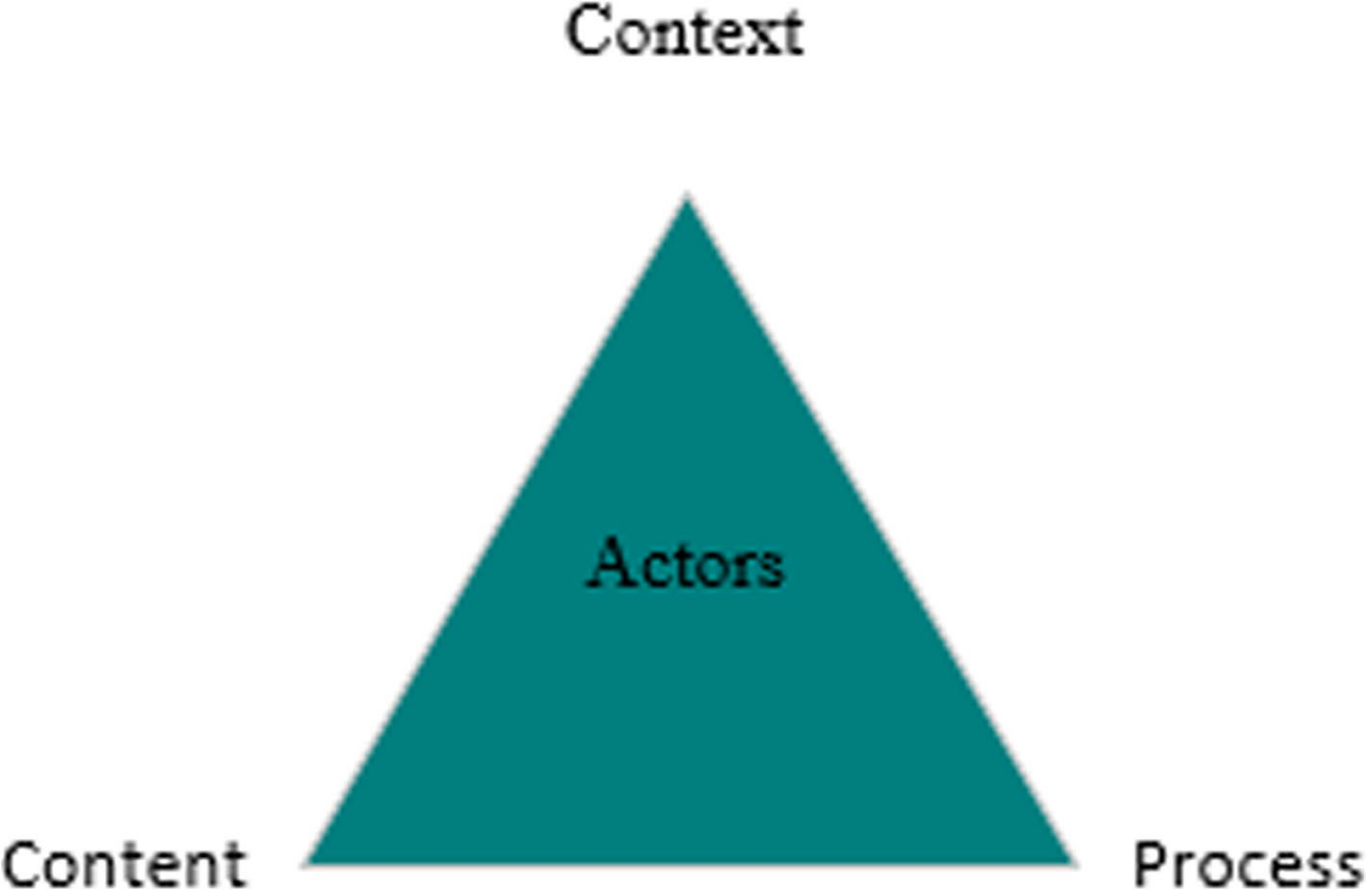
Policy Analysis Triangle framework, taken from Walt and Gilman [16]

To complement the ADEPT model, we drew on the “Policy Triangle Framework” devised by Walt and Gilson [16] in order to consider some of the factors (content, context, process, and actors) that impact policy development that are more relevant to low-and middle-income countries. We considered drawing on the concepts of the Health Policy Triangle during the data analysis, in order to complement and expand upon the ADEPT model. The triangle model is a useful model for analyzing a variety of health issues, including CHEs and impoverishment issues (see Fig. 2).

Individuals, institutions, and organizations that are stakeholders in this issue at hand can all be policy actors, with varying degrees of power in policymaking [16]. We used stakeholder analysis in accordance with the “Policy Triangle Framework”. For the stakeholder analysis, we developed a matrix to display the stakeholders’ positions, power, and interests [34, 35]. For each stakeholder, we displayed the current level of knowledge and power and noted whether or not each stakeholder supported a national policy on reducing CHE. We rank each respondent on a five-point scale with 1 as the minimum and 5 as the maximum [34, 35]. Then, we adjusted self-reported power by using the interpretation of other stakeholders as to the level of influence of the others to increase the validity of the scale. Also, we adjusted these judgments based on secondary data from the documents we reviewed [36].

### Analysis

The interview sessions were recorded digitally using two audio recorders, and the interviewer took handwritten notes conscientiously during the interviews. All the anonymously recorded files were transcribed verbatim by M.H. Thematic analysis was used to analyze the collected data [27]. The first author was primarily responsible for the analysis of the data, but all authors were involved and contributed to the analysis. Thematic analysis with an deductive approach was conducted to analyze the data [37]. During the analysis, data were systematically examined to identify repeated patterns of meaning. This process was composed of Braun and Clarke [47] six-step method: (1) familiarizing the self with the data, (2) generating initial codes, (3) searching for themes, (4) reviewing the identified themes, (5) defining and naming the themes, and (6) preparing the report [37]. During a series of meetings, researchers presented their findings and compared their individual categorizations with each other. Interpretations of these findings were discussed until a joint understanding was achieved [37]. Coding was done using the software MAXQDA 12.

The participants did not provide feedback on the findings. Therefore, to remove any potential risk of bias, there was critical reflexivity throughout the data collection and analysis phases. Moreover, to ensure trustworthiness, the categorization was discussed within the research team until a consensus was reached. Several methodological choices have been made to accomplish credibility and dependability such as peer debriefing, data and method triangulation, as well as the long-term immersion of the first author in this field and engaging four authors in the analysis process. Furthermore, the data obtained were again presented to a round-table discussion (constituting five senior policymakers) to enhance the validity and comprehensiveness of the study. The round-table discussion lasted approximately 2 h.

### Ethics

The Ethics Review Committee of Iran University of Medical Sciences (IUMS), Tehran, Iran (IR.IUMS.REC.1399.717). Provided the ethical approval for this study. Moreover, a signed written informed consent form was received from all the participants.

## Result

The findings are presented in two parts. The first part consists of the analysis of the identified policy documents according to the ADEPT model to determine the policy process and implementation. The second part contains the analysis of the policies for reducing CHEs using the policy triangle framework to determine policy development. Thereby, the findings were classified under 8 themes and 31 sub-themes extracted by document review, and 4 themes and 11 sub-themes extracted through interview.

### Document analysis

First, we identified important and relevant policy documents based on a review of the literature and media. Then, several policy documents were found that were directly or indirectly related to CHEs reduction in Iran. Ultimately, of all the policy documents yielded, 18 were retained. Table 1 lists all documents identified, along with a brief description of each. The establishment of a fifth Economic, Social, and Cultural Development Plan (2010–2015) by the Iranian Majlis that emphasized equity in healthcare and the reduction of household healthcare expenditure was one of the major transition in the Iranian health system. The launch of the HTP in 2014 designed by the MoHME based on the fifth 5-year health development national strategies to achieve UHC and reduce OOPs was another important transition in the Iranian health system.

**Table 1:** List of policy-related documents identified

No	Year	Policy document	Explanation of the document
1	1979	Article **29** of the Constitution	Emphasis on universal health insurance coverage
2	1980	The Law on Regulation of Health Care expenditures	The obligation of the MOHME to carry out the necessary studies within two months for the correct and fair regulation of medical and health expenses and to implement the relevant regulations in a timely manner
3	1984	Primary healthcare (PHC) system	The establishment of a PHC system through the National Health Network was one of the major transitions in the Iranian health system to achieve equity of financing and utilization
4	1994	The Public Medical Service Insurance Coverage Act (PMSICA)	This law was the second most important reform that provides formal health insurance coverage to several target populations (e.g. civil servants, people with disabilities, village dwellers, and nomadic tribes)
5	1995	Executive Regulations of Article **7** of the Public Insurance Law	Organizations were allowed to enter into contracts with medical centers to ensure the health of their employees
6	2000	Article **192** of the Third Development Plan Law	Moving toward UHC by the establishment of a surveillance system and preparedness for rationing services and referral system implementation to provide all health services free by the government
7	2002	The law of organization of health and treatment	The obligation of Iran’s government to close the annual budget from the beginning of 2003 in such a way that the grounds for the implementation of the UHC are formed and empowering people through self-employed insurance
8	2004	Regulation for social insurance of villagers and nomads	Establishing an insurance fund and covering more than 300 thousand villagers
9	2005	Fourth Development Plan Law (Article **90**)	For the first time, clear targeting was done on the issue of equitable (or fair) financing for health care. "Fairness in household financial contribution index (FFCI)" should be increased to 90%, people's share of health expenses should not increase from thirty percent 30%, and the number of impoverished households due to unaffordable health expenses should be reduced to 1%
10	2006	Communicating the general policies of "health" by the Supreme Leader of Iran (paragraphs 9 and 10)	Quantitative and qualitative development of health insurance and providing sustainable financial resources in the health sector
11	2007	Executive Regulations, Article **91** of the Fourth Development Plan	This article oversees the implementation of the family physician program in the country's health service delivery system
12	2008	Executive Regulations, Article **90** of the Fourth Development Plan	All government hospitals are obliged to provide all the supplies, equipment and medicine needed by the patients, and the patient is only responsible for the hospitalization deductible
13	2010	The Law of Targeting Subsidies	The implementation of this law has led to an increase in the costs of the health sector
14	2011	Article **38** of the Fifth Development Plan	The issue of reducing people's share of the health expenditures has been emphasized in a more complete way and with the same targeting of the fourth plan
15	2012	Family physician program	The urban family physician program was implemented in some parts of the country with extensive advertising and the full support of the Minister of Health
16	2013	Statute of Iran Health Insurance Organization	The first step was to implement Article 38 of the 5th National Development Plan and consolidate the country's insurance funds
17	2014	A collection of health transformation plan programs	This program aims to reduce out-of-pocket payments and increase the quality of hospital services at the level of Ministry of Health hospitals. Allocating appropriate credit from the government to this program is one of its key points
18	2017	Article **78** of the Sixth Development Plan	Reducing the percentage of households exposed to CHE through the extension and promotion of social health insurance to 1% and reducing OOP to 25%

**Fig. 3:**
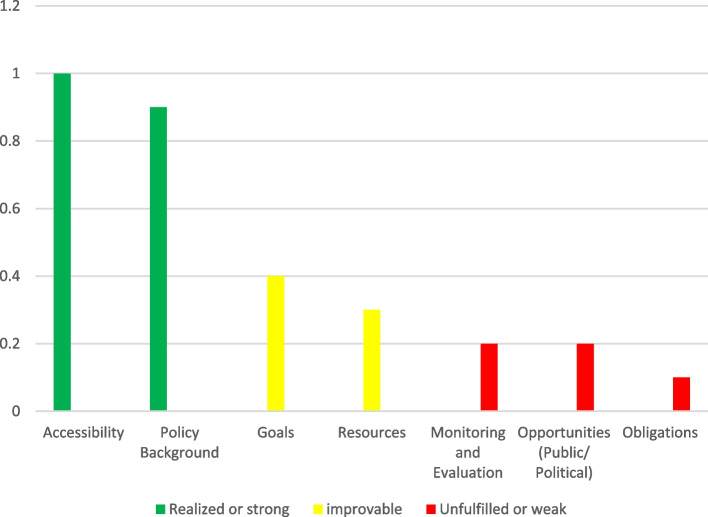
The results of the policy documents analysis by ADEPT model

In this section, findings from document review are presented under eight themes (document accessibility, policy background, goals, resources, monitoring and evaluation, Public Opportunities, Political Opportunities and obligations) and 31 Sub-themes that was developed and presented in a Table 2. Criteria were considered ‘Fulfilled/Strong’ if all the mentioned criteria were addressed, ‘Room for improvement’ if some criteria were addressed, and ‘Not fulfilled/Weak’ if no criterion was addressed (Fig. 3).

A Accessibility

**Table 2:** Definitions of the ADEPT criteria used to code data from the collected documents

Criteria	Definition
**Accessibility**	
1. The policy is accessible (hard copy and online)	Evaluated during the data collection stage—online availability was enough to satisfy this requirement
**Policy Background**	
1. The scientific grounds of the policy are established	The policy includes a discussion of health financing. The share of out-of-pocket payments (OOP, both formal and informal) in Total Health Expenditure (THE) and Measuring incidence and intensity of catastrophic payments are made explicit
2. The goals are drawn from a conclusive review of the literature	The policy shows evidence that the literature was reviewed, and this literature review was used in the decision-making process
3. The source of the health policy is explicit (Authority, data analysis, deduction)	The policy references a reputable source such as the World Health Report 2000 and draws on scientific studies such as the national health account, OOP index, and other health indices. Also, important documents, including the constitution and general healthcare policies, have predicted such a policy
4. policy encompasses some set of feasible alternatives	The policy describes potential alternative solutions to those that are intended to be implemented. For example, CHE reduction by increasing enrolment in government health insurance; compulsory membership, increasing financial stability through stable government subsidies, and increasing the government share of spending on health are stated in policy documents
**Goals**	
1. The goals are explicitly stated	The policy clearly states the overarching aims the policy program seeks to achieve; reduction of the OOP proportion of THE to 30%, and the reduction of households’ exposure to CHE to 1%
2. The goals are concrete enough to be evaluated later	Quantitative targets or benchmarks are built into the goal, as well as a time frame within which it is to be achieved
3. The goal is clear in its intent and in the mechanism with which to achieve the desired goal	Each goal is not accompanied by specific strategies or action items that can help achieve this goal once implemented
4. The action centers on improving the health of the population	Each goal in the policy is relevant, either directly or indirectly, to improving health outcomes. For example, the policy links the goal of WHO to ensure that the cost of care does not put people at risk of financial catastrophe
5. The policy is supported by evidence of external consistency in logically drawing a health outcome from the goals and policy outcome	The policy doesn't describe the influence of policies from other countries or Inter-Governmental Organizations' documentation on decision-making
6. The policy is supported by internal validity in logically drawing a health outcome from the goals and policy outcome	The policy doesn't link the scientific evidence to the goals and strategies being proposed
**Resources**	
1. The cost of condition to the community has been mentioned	In the implementation regulations of Article 90 of the 4th Development Plan, it has been specifically mentioned that the information related to the health expenditure index should be prepared by the Iranian Statistics Center and the Ministry of Health and Medical Education (MoHME) and included in the annual budgets
2. Estimated financial resources for the implementation of the policy are given	Despite the emphasis of the program and the approval of the law, none of the documents related to the policy, a specific source for financing the implementation of the policy has not been identified. Annual budgets are not provided in this field either
3. Allocated financial resources for the implementation of the policy are clear	The policy doesn't estimate the amount of money available for implementing the policy, and the sources of this money (the government, NGOs and IGO donors, etc.)
4. There are rewards/sanctions for spending the allocated resources on appropriate programs	The policy doesn't describe either financial rewards for implementing the policy or financial sanctions for not implementing the policy
5. Human resources are addressed	A description of the equitable distribution of human resources needed for implementation isn't provided. Also, there isn't an assessment of the resources based on WHO recommendations
6. Organizational capacity is addressed	The policy describes the infrastructure in place for implementation; for example, the MoHME responsible for carrying out policy implementation is described
**Monitoring and Evaluation**	
1. The policy indicates monitoring and evaluation mechanisms	The policy doesn't clearly describe the method by which monitoring and evaluation of the policy is to proceed
2. The policy nominates a committee or independent body to perform the evaluation	The policy mentions a Statistical Center of Iran responsible for monitoring and evaluation, by providing statistics and information necessary to analyze health cost indicators and transfer them to annual budgets. In this context, it is mentioned in the executive regulations of Article 90 of the fourth plan
3. The outcome measures are identified for each of the explicit and implicit objectives	For each goal, there is a description of the indicators that are used to measure the progress toward this goal
4. The data, for evaluation, are collected before, during and after the introduction of the new policy	The policy doesn't report the baseline quantitative (or qualitative) data for each goal
5. Follow-up takes place after a sufficient period to allow the effects of the policy change to become evident	The policy doesn't describe the time periods within which evaluations of the policy implementation are to be conducted
6. Other factors that could have produced the change (other than policy) are identified	The policy doesn't consider social, economic, cultural, and other factors that could increase CHE rates that may fall outside the specific strategies that are implemented
7. Criteria for evaluation are adequate and clear	The policy doesn't describe the method for collecting and evaluating data to obtain specific outcome measures
**Public Opportunities**	
1. Multiple stakeholders are involved	The policy names multiple individuals, groups, or organizations that have a role in decision-making or policy implementation, such as insurance organizations, MoHME, NGOs, and services providers,…
2. Primary concerns of stakeholders are recognized and acknowledged to obtain longer term support	The policy doesn't identify the primary concern of each stakeholder and doesn't take it into account in decision-making
**Political Opportunities**	
1. The political climate has either worsened or improved	The policy doesn't describe the political factors that may have influenced decision-making and how they have changed over time
2. Cooperation between public and private organizations has either worsened or improved	The policy doesn't describe the nature and extent of the cooperation between the public and private sectors of health care
3. The lobby for the action has either worsened or improve	Lobbying groups, their mandates, and the effect they have on decision-making aren't described
**Obligations**	
1. The obligations of the various implementers are specified—who must do what?	Each goal or strategy has a specific actor (individual or organization), but the responsibility for implementing the strategy is not specified
2. Scientific results are compelling for action	The policy doesn't express a clear obligation to act based on scientific results laid out in the document. Failure to implement the policy clearly indicates a lack of professional obligation to the policy. Due to the complexity of the issue and its intersectoral nature, organizations are not independent in the implementation of this policy and its strategies, and these interactions have sometimes created role interference

One of the themes was accessibility, which included evaluating the accessibility of the policy document in both hard copy and online formats. It was found that the policy was accessible through both formats, and online availability was deemed sufficient to meet the accessibility requirement. It is noteworthy that, core documents were accessible from the MOHME and IPRC websites.

B Policy background

The policy has established the scientific grounds for health financing and has clear goals derived from conclusive literature reviews. The policy references reputable sources, including the World Health Report 2000, and has identified potential alternatives for policy implementation. The sources of the policy are explicit, including the constitution and general healthcare policies. Finally, the policy describes feasible alternatives to achieve its intended goals.

III Goals

The policy's goals are explicitly stated and are concrete enough to be evaluated later, with quantitative targets and a time frame. Each goal has specific strategies or action items to achieve the desired outcome, centered on improving the health of the population. However, the mechanism used to achieve the desired goals has not been clear. Also, the policy lacks evidence of external consistency from policies of other countries or inter-governmental organizations and does not link scientific evidence to the proposed strategies.

IV Resources

The policy mentions the cost of the condition to the community and the need to include health expenditure information in annual budgets. However, there is no estimation of financial resources for the policy's implementation, and no specific source of funding has been identified. Additionally, there are no financial rewards or sanctions for spending allocated resources appropriately, and the equitable distribution of resources isn't addressed. On the positive side, the policy does describe the infrastructure in place for implementation, with the MoHME responsible for carrying out policy implementation.

E Monitoring and evaluation

The policy doesn't clearly describe the method for monitoring and evaluating the policy implementation. However, the Statistical Center of Iran is mentioned as responsible for monitoring and evaluation. For each explicit and implicit goal, the policy provides outcome measures to evaluate progress, but baseline data and time frames for evaluations are not specified. Additionally, the policy doesn't address potential factors other than the policy that could influence outcomes, and there is no clear method for collecting and evaluating data for specific outcome measures.

F Public opportunities

It was found that multiple stakeholders have been involved in the policy-making process, including insurance organizations, the Ministry of Health and Medical Education (MoHME), NGOs, the private sector, and service providers. However, the policy does not identify the main concern of each stakeholder and does not take them into account in decision-making. Overall, the findings suggest that there is a need for greater stakeholder engagement and consideration of their concerns in policy-making.

G Political Opportunities

It was found that the policy does not provide a detailed description of the political factors that may have influenced decision-making or how they have changed over time. Additionally, the policy does not describe the nature and extent of cooperation between the public and private sectors of healthcare. However, due to the low-funded and low-quality health care of the public health system which forces consumers to seek care from the private sector, and also the high healthcare costs in the private sector, the economic burden of health spending mostly lies on private spending which in Iran is majorly by OOP expenses. Furthermore, the policy does not provide information on lobbying groups, their mandates, and the effect they have on decision-making. Overall, the findings suggest that the policy lacks a comprehensive analysis of the political opportunities and challenges that may impact policy implementation, and it may benefit from further consideration of these factors.

H Obligations

Based on the information provided, it seems that there is a lack of clarity in terms of the obligations of the different actors involved in implementing the policy. While the document may specify the goals and strategies, it does not clearly outline who is responsible for carrying them out. Additionally, the policy may not clearly state the obligation to act based on scientific results presented in the document. This lack of clarity may lead to a lack of accountability and responsibility for implementing the policy, which can hinder its effectiveness. Furthermore, the intersectoral nature of the policy may create role interference, making it difficult for organizations to independently implement the strategies. This highlights the need for clear communication and collaboration among the different actors involved in implementing the policy.

In general, in terms of the eight criteria that determine policy development and policy impact implementation, the set of programs related to the policy on reducing CHEs did not meet suitable conditions. The only document accessibility criterion, policy background criterion, and to some extent, the goals criterion influenced the policy process. Further, the resources, monitoring and evaluation, opportunities, and obligations criteria negatively affected the policy process and implementation. So, the CHE reduction policy was not successful in its process and implementation.

### Context, content, actors, and process in the development of the CHE reduction policy

In total, 35 interviewees, including policymakers, academics, practitioners, and members of civil society, agreed to participate in this study. In this section, findings from interviews were analyzed according to the policy triangle framework and presented under four themes including; context, content, policy process, actors, and 11 sub-themes including; Political, Situational, Structural, Cultural, Consistency of goals, Internal logic, Conflict of interest, Power arrangement, Stakeholder participation, Monitoring and evaluation, Stewardship and leadership.

#### Context

This study investigated the contextual factors influencing Iran's CHE-reduction policies. To facilitate understanding of the policy environment, we categorized contextual factors as ‘situational,’ ‘structural,’ ‘cultural,’ and ‘political’ because national public policies can be explained by these factors. The themes and subthemes are presented in Table 3.

**Table 3:** The effective factors in the failure of CHE reduction policies in the category of context

Theme	Sub-theme	Category	Subcategory
Context	Political	political atmosphere in Iran	Factionalism in health policy making
			The government's opposition to the fourth development plan
		Political decisions-making	Lack of political will to implement programs
			Lack of public demand
			Lack of priority for health in the governance perspectives
			Lack of support from political authorities
			The superiority of the political component over the technical component of the decision
	Situational	Economic factors	Inefficient economic system
			Economic impulses caused by sanctions
			Economic problems caused by the spread of covid-19
			Economic instability
			The decrease in gross domestic product per capita (GDP)
			The effect of targeted subsidies
			The high cost and large copayment for some medical services
			The high administrative costs of the departments
			Improper payment system
			The high inflation in the health sector
		Resource shortage problem	Domestic general government health expenditure (GGHE-D) as percentage of general government expenditure [46] (%)
			Planning without resource allocation
			Insufficient financial resources of the health sector
		Resource management	High priority for allocating resources to treatment
			Poor resource management
			Weakness of compliance with the referral system
	Structural	Service delivery structure	Inefficiency of the health insurance system
			Fragmentations in Iran’s health insurance system
			The inefficiency of the public sector in providing quality services
			Low financial protection against healthcare expenditures for the insured persons
			High coinsurance rates
			A notable rate of insurance coverage duplication
			Government entrepreneurship in the health sector
		Decision making structure	Concentration of policy-making, monitoring and implementation in one institution
			Improper organization of health care system components
			Incompetence of implementing organizations
			Weakness of the health system structure
			lack of reliable data and statistics
			Lack of transparency of responsibilities
	Cultural	Cultural factors	The culture of specialization care in Iran
			The culture of consumerism in health care in Iran
			The public demand for utilizing advanced technologies
			The paternalism and medicalization
			Changing people's lifestyles
			The lack of media in promoting CHE reduction policies
		Social factors	Consumer moral hazard
			Lack of real health literacy
			Lack of public demand
			Ignoring policy by the media
			Social determinants of health
			Supplier induced demand

Nearly half of the participants described the macro-environmental conditions of the country as unfavorable during the implementation of the policy on reducing CHE. The government was not in favor of the policies on reducing CHE and did not have much determination to design and present an alternative plan. Also, the findings do not show any indication of prioritizing health from the government's perspective during the study years. The most important contextual factor for the policies on reducing CHE was the lack of financial resources allocated for policy implementation. After the launch of the HTP in 2014, there was a serious move to reduce OOP but after 2016, there has been much fluctuation in OOPs due to the country’s economic conditions, including foreign sanctions, a new economic crisis associated with the COVID-19 epidemic, decrease in GDP per capita PPP, general government debt and domestic healthcare financing of the HTP. This issue was mentioned by most of the informants.

*“Health has never been the priority of the government and the government has never fully paid the health share of the budget. This has caused a delay in financing from insurance companies and an increase in the people's share of health payments.”*

#### Content

Findings revealed that the policy of protecting against CHEs and impoverishment is stated as a specific goal in the mega documents and has a specific international basis, but in terms of its specific objectives, mechanisms, strategies, and especially supporting documents such as executive regulations, it was weak.

*“The plans were very idealistic and far from reality, and the country's economic power and capacity were not taken into account.”*

Another interesting point from the point of view of the experts is the deviation from the main policy in the regulations and instructions related to the policy. According to investigations, the executive regulations of Article 90 of the Fourth Development Plan were drafted two years after the plan was approved and approved one year later. From another point of view, the contradictions in some executive programs with the goals of the program, inadequate rules, and conflicting or non-aligned rules have been stated as factors in the policy not being properly implemented (Table 4).

*“There is no executive policy and mechanisms for reducing out-of-pocket payments that can be used as supporting documents for legal regulations.”*

**Table 4:** The effective factors in the failure of CHE reduction policies in the category of content

Theme	Sub theme	Category	Subcategory
Content	Consistency of goals	Targeting	Targeting has been ideal
			Failure to clearly state goals
			Weakness in supporting documents
		Technical requirements of the application	Failure to implement legal mechanisms
			Lack of budget support for politics
			Inconsistency in the rules
	Internal logic	Prioritize	Lack of priority of the health sector in the government's view
			Sectoral perspective on program design
			The program is paper-based
			The populist point of view of senior managers to issues
			Competition between programs and commitments to attract resources
		The nature of politics	This policy is imported as an international policy
			The populist nature of this policy
			Incorrect program defaults
			Lack of serious attention to people’s needs
			Imposing the need for the government by experts

#### Actors

**Fig. 4:**
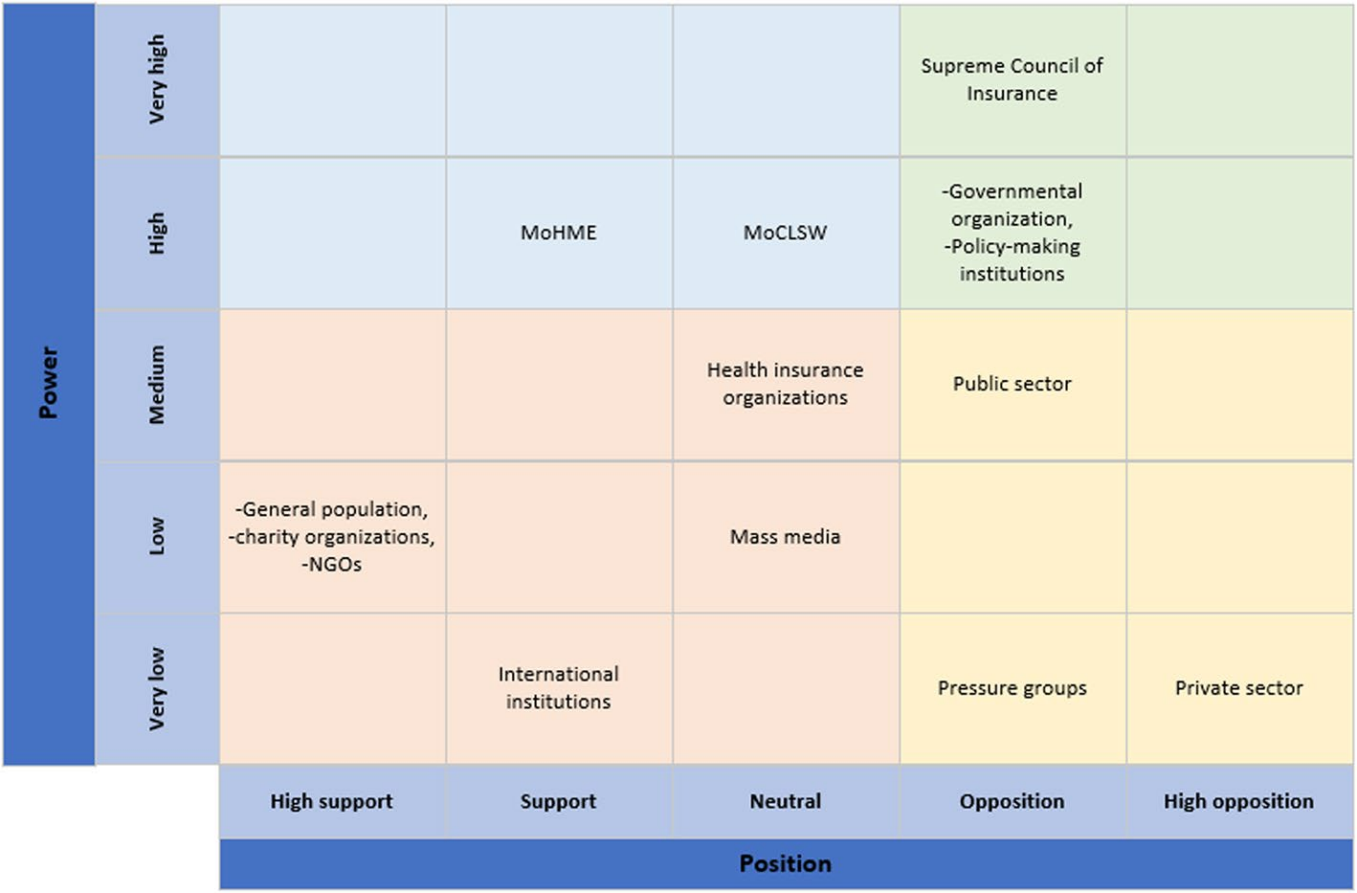
A power & position analysis

We present stakeholders’ roles, interests, knowledge levels, self-reported power (adjusted), and their positions in Supplementary 4. In Iran's health sector, the power and position of key political actors play an important role in the implementation of policies. We map the estimated power of each stakeholder based on their position to indicate their potential influence (Fig. 4, force-field mapping).

Although gaining support from all stakeholders in the early stages of development, implementation, and evaluation of policies is an important factor in achieving the goals of those policies, but this importance has been neglected in the policy on reducing CHE. The study also revealed that an important challenge in reducing CHEs is the dominance of a top-down approach in formulating and implementing policies, which affects the relationships between actors.

*“Both top-down and bottom-up approaches have not been employed in formulating and implementing policies. The use of a top-down approach alone can be seen as ineffective in addressing the complex and nuanced issues involved in reducing CHE, as stakeholders may have different perspectives and priorities. Similarly, a bottom-up approach that only involves those directly affected may not fully consider the broader implications of the policy. A more inclusive and participatory approach that incorporates both top-down and bottom-up perspectives, and establishes a dialogue among all stakeholders, could be more effective in addressing this.”*

Also, the presence of physicians in policy-making authorities and policy implementation, such as the Parliament's Health and Treatment Commission, the MOHME, the Medical System Organization, and even insurance organizations, as well as in the health market and the private sector of the health system, can cause conflicts of interest in policy implementation and resource allocation.

*“The mastery of the physician profession on decisions is definitely very important.”*

The findings show that MoHME had the direct impact on the policy of reducing CHE, with the most support and power. Unfortunately, Iran's chaotic health system, lacking strong stewardship and fragmentation in the financing system, as well as a strong conflict of interest in policy formulation and implementation, has predisposed to decreasing accountability and increasing corrupt activities. Although the MoHME is in charge of stewardship of health systems in upstream policies, in addition to health policymaking, service delivery and, in some cases, financing are carried out simultaneously in this organization. So, the strategic decisions are made largely by consensus among experts, who have the greatest influence on increasing OOP. Healthcare providers and suppliers are the most powerful stakeholders that play a more prominent role, with an indirect impact on CHE rates.

*“There is a huge conflict of interest in the Ministry of Health. The Minister of Health and the Deputy Ministers and most of their experts are physicians and their hands are in the health market. This prevents the implementation of approved laws.”*

*“The Ministry of Health's role is stewardship and it should define the guidelines, but when it acts as a service provider, it is certain that it works in such a way that its hospitals are managed well and their debts are paid, so it implements as a presentation the health service provider, not the health stewardship.”*

In this study, the effect of groups and individuals on policy implementation is depicted as a conflict of interest and the effect of organizations as a power arrangement (Table 5).

**Table 5:** The effective factors in the failure of CHE reduction policies in the category of actors

Theme	Sub theme	Category	Subcategory
Actors	Conflict of interest	Clinical perspective in Iran health system	The presence of the majority of physicians in all policy making and decision-making authorities
			Physician authority
			Professional bias of physicians in legislation and implementation
		Model of policy making	Lack of a unique decision maker stewardship for health system
			General policy patterns in the country
			Priority of specialized and curative care in policy making
			Ignoring the scientific bases of policy making
	Power arrangement	Health service provider behavior	Healthcare provider profitability
			Improper distribution of power
			System design based on the interests of service providers
			Strong provider lobby
		Health market behavior	The difference in payment in the public and private sector of the market
			The role of the market in increasing induced demand
			Expansion of informal payments
			Privatization in the health system
			The inability of the government to regulate the health market
			Non-participation of the provider in the health market risk

#### Process

The MoHME established the policy in steps to protect people from CHEs and poverty. In this regard, the reform entitled “HTP” was designed by MOHME to reduce CHE for inpatient services at the hospitals affiliated with MOHME. Political will and stakeholder position are critical for successful policy implementation and securing financial support from powerful actors. However, in the CHE reduction policy formulation, the lack of participation of key stakeholders in the design of policies and the lack of transparency of the duties and responsibilities of different departments decreased the level of precision in policy implementation.

*“One of the most important planning problems in Iran is that we do not specify who should be responsible for what. In the policy of reducing catastrophic health expenditures, despite the passage of years since the approval of the policy and the failure to achieve the goals of the policy, it is not clear who should answer and who should be asked for an answer.”*

In addition, inappropriate inter-sectoral collaboration, a lack of comprehensive communication strategies to articulate the policy, and the absence of a stakeholder management strategy were supposed to be the main factors that led to the failure of policy implementation. Monitoring and evaluation mechanisms are an important part of the policy process because they increase the credibility of policy analyses. The participants considered the weakness of regulatory institutions, the lack of accountability of organizations, and the lack of transparency of the policy monitoring mechanism as the most important obstacles to the effective implementation of the policy on reducing CHE. The main source of health governance failure in Iran was weak governance, whose roots lay in a lack of stewardship. The weakness of the stewardship function of the MoHME influences the policy on reducing CHE through regulation.

*“Roles are not well defined and the relationship between roles and organizations is not clear. The role of the Ministry of Health is stewardship, but it plays the role of a health service provider. Also, there is poor coordination and communication between organizations and departments.”*

There are three main challenges in stewardship that result in financial hardship for Iranian households, including lack of commitment, weak internal governance, and weak external leadership (Table 6).

**Table 6:** The effective factors in the failure of CHE reduction policies in the category of process

Theme	Sub theme	Category	Subcategory
Process	Stakeholder participation	Importance of stakeholders	Non-participation of stakeholders in policy design
			Non-acceptance of responsibility between stakeholders
			Non-alignment of stakeholders in the program
		Intersectoral collaboration	Improperly implement program mechanisms
			Weak intersectoral coordination
			Disagreement of various stakeholders with the program
			Improper organization of health system components
	Monitoring and evaluation	Monitoring and evaluation	Weakness of regulatory institutions
			Uncertainty of policy monitoring authority
			Selective enforcement of rules
			Weak oversight of the entire health system
			Weakness in parliamentary oversight
	Stewardship and leadership	Internal governance	Lack of determination in implementing programs
			Managers are not familiar with the subject
			Managers' short-term attitude to solving problems
			Weakness in the stewardship of the MoHME
			lack of commitment
			Management instability (change management)
		Interdisciplinary governance	Weakness in leadership and governance of the health system
			Lack of transparency of duties
			Lack of interaction between policy-makers and executives
			Gap exists between policy formulation and implementation

**Fig. 5:**
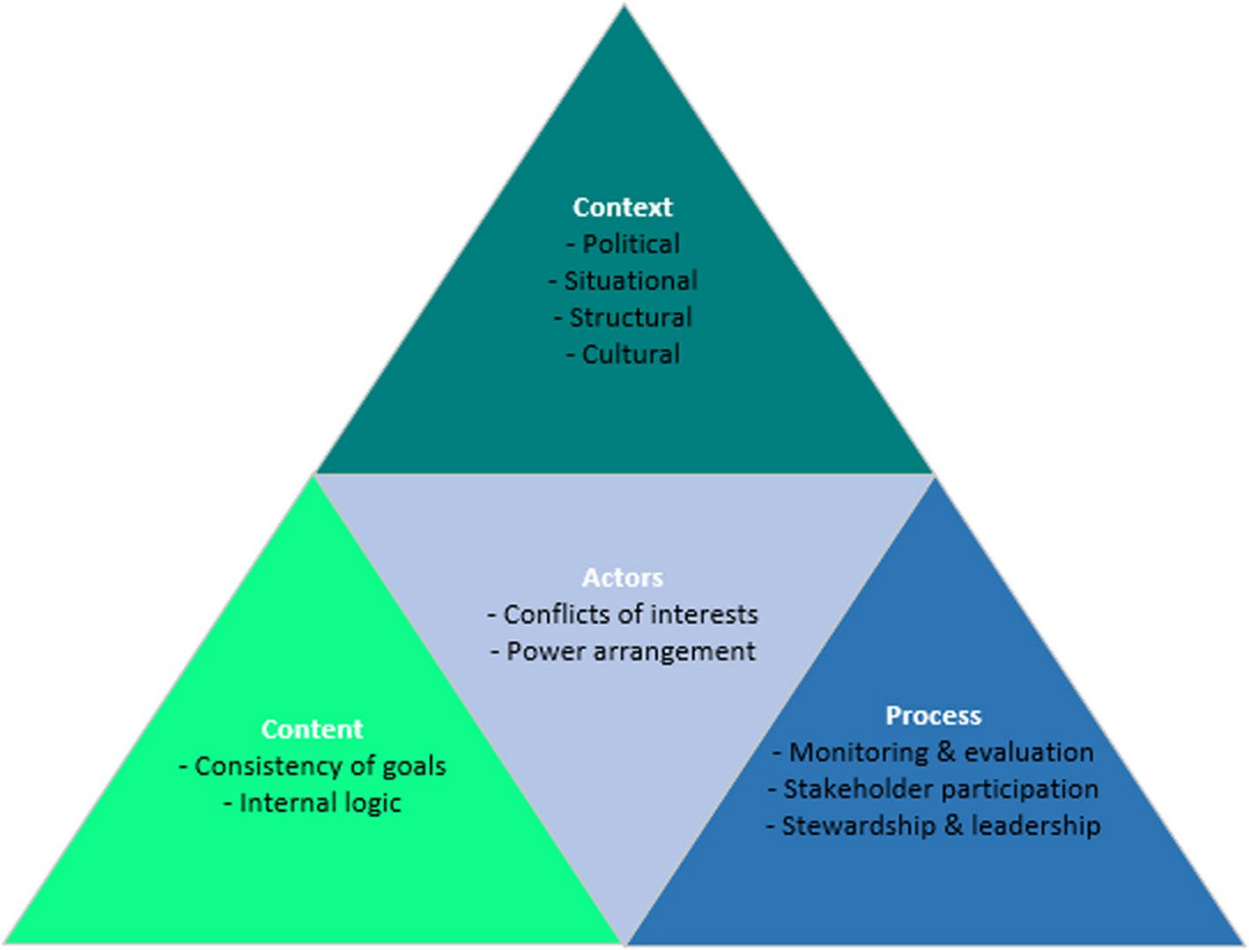
Categorization of factors affecting the policy of reducing CHE in Iran based on analysis triangle model

Therefore, the findings from interviews with experts in policies to reduce households' exposure to CHEs and impoverishing using Walt and Gilson's policy triangle method in four categories were show in the Fig. 5.

## Discussion

This qualitative study was conducted to critically analyze the current policies for reducing catastrophic health expenditures (CHE) and impoverishment in Iran. The ADEPT model and Walt and Gilson's analysis triangle model were applied as analytical frameworks to analyze the data gathered through review political documents and semi-structured interviews with key informants.

The thematic analysis of policy documents delineated that document accessibility, clear statement of goals, and policy background criteria positively influenced the policy process, as they were in the public interest and supported by the government and MoHME. But, the Monitoring and Evaluation, obligations, opportunities (political, public, and media), and resources criteria negatively affected the policy process and implementation.

The strategies, activities, and mechanisms involved with implementing the policy on reducing CHEs, and the actors responsible and their obligations for each activity had not been shown in national policy documents. The result of the policy analysis in Ethiopia shows that reducing CHE needs to establish a new financial protection mechanism by developing supportive strategies [38].

Also, high-quality scientific evidence didn't use in policy-making and problem-solving. According to the evidence the development and implementation of the Health Transformation Plan (HTP), one of the most important national health action plan in Iran, has many challenges in its continuity as was not based on scientific evidence [39, 40]. Another study shows that the Healthy China 2030 Plan that set targets for the percentage of the OOP payment in total health expenditure to monitor the level of health financial risk protection had many challenges as was not based on scientific evidence [41]. Increasing awareness of the need for evidence-based health policies demonstrates the importance of health research in developing health policies. So, Strengthening the links between policy and research will enhance accountability, to ensure that technical design is appropriate to the context and that policymaking is informed by robust evidence.

The parliament and the government had not approved the legal and administrative facilities as well as sufficient specific resources to implement the policy. Also, decrease in GDP per capita PPP, the economic and political instability impaired the sustainability of financing during the policy implementation years. Analysis of the HTP revealed that a lack of adequate resources and low governmental budgets have led to failure in policy implementation and a significant dependency on people’s own pockets [42]. The inadequate budgetary allocation to healthcare has significantly influenced recurrent and capital health expenditures and increased OOP in Nigeria [43]. Consequently, the findings of this study reflect the necessity of the government as far as possible to increase public financing on health in order to strengthen healthcare system efficiency against households OOP spending for necessary healthcare utilization.

This study demonstrated that decision-making on reducing CHE required the participation of all stakeholders to reach a consensus. The policy named multiple individuals, groups, or organizations that have a role in decision-making or policy implementation, but didn't identify the main concern of each stakeholder and didn't take it into account in decision-making. So, the CHEs reduction policy has not resulted in the desired policy outcomes. Both top-down and bottom-up approaches have not been employed in formulating and implementing policies. While establishing a dialogue or policy discourse to achieve interactive and inclusive policy-making could address the weaknesses of this policy [44, 45]. Russell et al. offer the only solution to tackling the problems associated with rapid growth in OOP in Australia by the involvement of all stakeholders including governments at all levels, providers, industry and consumers in priority decisions at a policy level [46].

A host of factors of various natures were identified through the health policy triangle model that impeded favorable CHE reduction policies implementation. This study highlights the important role of “conflicts of interest,” “contextual factors”, “intersectoral relationships”, and “monitoring and evaluation”, in CHEs reduction policy during the years of study in Iran. The “conflicts of interest” were a central factor due to their interaction with other factors.

The findings showed that throughout the country's political, social, and economic environment during the years of study, the behavior of the main stakeholders toward politics has been influenced by personal and organizational conflicts of interest. However, it seems that political will is not present among politicians to implement the policy on reducing CHEs. Other studies in Iran showed that to achieve the goals of UHC, conflicts of interest were identified as the leading factors on the way to achieving those goals [47–49]. So, the MoHME needs to overcome its longstanding conflicts of interest in health policy-making, all in line with upstream policies and laws, along with its rather long and winding road to achieve UHC, as well as enhancing transparency and responsiveness of the health system in Iran.

This issue, along with other contextual factors such as the lack of political commitment of the government to implement the policy, social determinants of health, economic instability caused by sanctions and inflation, and the lack of effective support of civil society, meant that despite the failure to achieve all goals of the policy to reduce CHEs, there was no special responsibility for any of the stakeholders. Similar studies of policy analysis in Iran acknowledged the importance of contextual factors in policy implementation [50–53]. This finding is consistent with Margaret E Kruk et al.’s study [54]. Furthermore, achievement of the UHC goals and decrease exposure to CHE will require effort from good leadership and management competencies and Strong regulatory mechanisms with transparency through good monitoring, measurement, and reporting practices, but begin with a political commitment from heads of state and ministers [54].

The findings indicated that intersectoral collaboration is critically important in policy-making and policy implementation and leads to improved effectiveness. More than half of the interviewees believed that the intersectoral collaboration of all non-health organizations and public–private participation in the policy process of reducing CHEs in Iran was insufficient. Despite the fact that intersectoral collaboration and public–private partnership are one of the main pillars of achieving the goals of UHC [55, 56] and also in health policy-making and the process of policy implementation [57]. Therefore, given that the most important social determinants of health are outside the health sector, the collaboration between this sector and other areas can provide a supportive context for equitable financing and decrease exposure to CHE [58].

Timely monitoring and evaluation of policies are an essential part of the policy cycle, as they can facilitate evidence-based policy design and implementation, increase the policy's accountability and transparency, demonstrate achievements toward policy objectives, and assess the policy's effectiveness, efficiency, results, and impacts [59]. Results of the current study showed that the weakness of regulatory institutions in monitoring and evaluating the CHEs reduction policy was one of the obstacles to implementing this policy in Iran. While monitoring and evaluation of the policy can provide many lessons for health system policy-makers. Similarly, Yousefi Khoshsabegheh et al. found that the most important facilitators for policy implementation were monitoring and evaluation [60]. A scoping overview of reviews that was done by Dominika Bhatia et al. demonstrated that lack of process evaluations and monitoring; lack of comparable and standardized measurement and short follow-up periods were the main gaps to achieving financial risk protection as an indicator of Sustainable Development Goal 3 universal health coverage target in low-income and middle-income countries [61].

These findings have contributed to the persistence of CHEs and impoverishment, hindering the progress toward UHC in Iran. To overcome these challenges, it is crucial for the government to increase political will and commitment to UHC, strengthen intersectoral collaboration and stakeholder engagement, and also, enhance the role of regulatory institutions in monitoring and evaluating health policies. Future policies and strategies aimed at reducing CHEs and ensuring UHC in Iran should be informed by robust scientific evidence and involve a collaborative and participatory approach with all relevant stakeholders. In addition, future studies and scenario-based planning can provide valuable insights into potential challenges and opportunities in the future, allowing health policymakers to make evidence-based decisions and take proactive measures toward providing access to equal health care for all.

## Study strengths and limitations

Prior to this study, the ADEPT model, which explains policy successes and failures, had not been used in Iran. The policy triangle framework used for analysis helped build a comprehensive understanding of the policy process. As for limitations, the occurrence of recall bias is one. However, the influence of recall bias on the study results was minimal since the data from the interviews on the CHEs reduction policy were validated by the document review. The respondents interviewed may not be a representative sample of all stakeholders that could influence the policy process. Other limitations of the present study were the participation of only Iranian experts and the document review, which was limited to research conducted in Iran, which could reduce the generalizability of the results. To increase the generalizability and validity of the study results, the researchers tried to identify and categorize the factors using several different methods (a qualitative study including document review and semi-structured interviews, and an expert panel).

## Conclusion

This study assessed the status of the CHEs policy process and implementation and the factors of policy failure in Iran. The study collected data on determinants of the policy process and performance of the health sector from national stakeholders using key informant interviews. Multiple stakeholders’ involvement and their underlying values and ethical perspectives indicate the complexities of policy-making to achieve a reduction in catastrophic and impoverishing health expenditures in Iran. The correct implementation of the policy depends upon strategies to improve intersectoral collaboration through setting common goals and creating a shared vision; strengthening the stewardship and accountability role of the MoHME; designing policy monitoring and evaluation mechanisms; drafting a document to prevent personal and organizational conflict of interests; participation of service providers in the policy-making process to increase commitment; increasing political will; and media support. In conclusion, achieving UHC in Iran requires a comprehensive approach that considers multiple factors and stakeholders, as well as proactive planning and decision-making, which has not been achieve yet due to the mentioned challenges. Only after overcoming these obstacles, Iran health system can successfully reduce CHEs, improve health outcomes, and provide equitable access to equal health care for all.

It’s crucial to have a robust and long-term planning strategy to anticipate changes in the healthcare sector and make evidence-based decisions to improve the overall health outcomes in Iran. So, health policymakers need to be equipped with new tools to face the future of health expenditures, anticipate changes, and make appropriate policies for excellence. Future studies and scenario-based planning in the context of health expenditures in Iran can help to effectively plan and implement policies towards achieving UHC goals and reduce CHE in Iran.

## Supplementary Information

**Additional file 1:** **Supplementary 1.** Interview questions.

**Additional file 2:** **Supplementary 2.** Characteristics of participants.

**Additional file 3:** **Supplementary 3.** ADEPT criteria associated with each determinant of policy impact.

**Additional file 4:** **Supplementary 4.** Analysis of Iran stakeholders’ interest, knowledge level, power, and position. 

## Data Availability

All data generated or analyzed during this study were included in the published article.

## Abbreviations

CHE: Catastrophic health expenditures
ADEPT: Analysis of Determinants of Policy Impact
OOP: Out-of-pocket
WHO: World Health Organization
UHC: Universal Health Coverage
SDGs: Sustainable Development Goals
GNI: Gross national income
HTP: Health Transformation Program
NDP: National Development Plans
THE: Total Health Expenditures
MoHME: Ministry of Health and Medical Education
SSO: Social Security Organization
IHIO: Iran Health Insurance Organization
EMRO: Eastern Mediterranean Region Office
