# Supplementary material for: Policy analysis of the protection of Iranian households against catastrophic health expenditures: a qualitative analysis
Source: BMC Health Serv Res. 2023 May 5;23:445. doi: 10.1186/s12913-023-09275-0 (PMC10161991; doi:10.1186/s12913-023-09275-0)
Supplement: Supplementary file 1 — Additional file 1: Supplementary 1. Interview questions. [file 12913_2023_9275_MOESM1_ESM.pdf]

Supplementary 1. Interview questions

1. Has your organization participated to an acceptable extent in the design and development of the policy?
2. Was the intersectoral collaboration of organizations related to policies desirable?
3. At the time of designing the policy, were the priorities of each of the stakeholders considered to attract their long-term cooperation?
4. Is the key decision-making process in Iran's health system based on examining the relationships between key actors?
5. Has the cooperation between decision-making and policy implementation levels been appropriate?
6. Were the goals related to the implementation of the policy clearly stated?
7. Were the goals achievable according to the country's contextual conditions?
8. Were the executive regulations clearly defined?
9. Were the goals quantitatively or qualitatively assessable?
10. Were the indicators and policy monitoring and evaluation tools clearly stated?
11. Has the process of allocating resources to the health system in the country been fair and appropriate?
12. Have specific agencies or groups supervised the implementation of the policy?
13. Were the results of policy implementation evaluated and given feedback to executive bodies and policy makers?
14. Were there specialized human resources to implement the policy in organizations?
15. Were the rules and regulations necessary to implement the policy and achieve the goals of the policy formulated?
16. Were the duties of each organization in relation to the implementation of the policy clearly stated?
17. Was there the necessary executive capacity and power in the country's health system to implement the policy?
18. Has the focus of resource allocation been on the policy of reducing CHE?
19. Could you walk us through the major steps of the development process of the policy? (process)
20. Have there been consultation processes? If so, what did they look like? (process/content)
21. Why Iran has not been successful in implementing the policy of reducing CHE? (process)
22. Which reasons and developments do you consider important drivers for the development of the policies on reducing CHE? (process)
23. Which reasons and developments do you consider important drivers for the failure of the policies on reducing CHE? (context)
24. How do you analyze the content of this policy?
25. Which actors do you consider to have made a relevant impact on the initiation and development of the policies on reducing CHE (actors)
26. Did the policymakers and policy implementers have the necessary power to play the right role?
27. How were the goals of the policy selected? (context/content)
28. What have been the effects of the policies? (effects)
29. What were the strengths and weaknesses of the policy-making process?
30. Between the two factors of lack of resources and resource management, which one played a greater role?
