# Supplementary material for: Policy analysis of the protection of Iranian households against catastrophic health expenditures: a qualitative analysis
Source: BMC Health Serv Res. 2023 May 5;23:445. doi: 10.1186/s12913-023-09275-0 (PMC10161991; doi:10.1186/s12913-023-09275-0)
Supplement: Supplementary file 2 — Additional file 2: Supplementary 2. Characteristics of participants. [file 12913_2023_9275_MOESM2_ESM.pdf]

Supplementary 2. Characteristics of participants

**participants**		**No.**
Affiliation	Parliaments	4
	Public health care	4
	Private health care	4
	IHIO	7
	MoHME	8
	Faculty member	8
Age	< 50	16
	>50	19
Gender	Men	30
	Women	5
