# Supplementary material for: Policy analysis of the protection of Iranian households against catastrophic health expenditures: a qualitative analysis
Source: BMC Health Serv Res. 2023 May 5;23:445. doi: 10.1186/s12913-023-09275-0 (PMC10161991; doi:10.1186/s12913-023-09275-0)
Supplement: Supplementary file 3 — Additional file 3: Supplementary 3. ADEPT criteria associated with each determinant of policy impact. [file 12913_2023_9275_MOESM3_ESM.pdf]

Supplementary 3. ADEPT criteria associated with each determinant of policy impact

Determinant	Criteria
Accessibility	1. The policy is accessible (hard copy and online)
Policy background	1.The scientific grounds of the policy are established<br>2. The goals are drawn from a conclusive review of literature<br>3. The source of the health policy is explicit<br>a. Authority (one or more persons, books, scientific articles or sources of information)<br>b. Quantitative or qualitative analysis<br>c. Deduction (premises that have been established from authority, observation, intuition or all three)<br>4. The policy encompasses some set of feasible alternatives
Goals	1. The goals are explicitly stated<br>2. The goals are concrete enough to be evaluated later<br>3. The goal is clear in its intent and in the mechanism with which to achieve the desired goals<br>4. The action centers on improving the health of the population<br>5. The policy is supported by evidence of external consistency in logically drawing a health outcome from the goals and policy outcome<br>6. The policy is supported by internal validity in logically drawing a health outcome from the goals and policy outcome
Resources	1. Financial resources are addressed<br>a. The cost of condition to community has been mentioned<br>b. Estimated financial resources for implementation of the policy is given<br>c. Allocated financial resources for implementation of the policy are clear<br>d. There are rewards/sanctions for spending the allocated resources on appropriate programs<br>2. Human resources are addressed<br>3. Organizational capacity is addressed
Monitoring and Evaluation	1. The policy indicates monitoring and evaluation mechanisms<br>2. The policy nominates a committee or independent body to perform the evaluation<br>3. The outcome measures are identified for each of the explicit and implicit objectives<br>4. The data, for evaluation, are collected before, during and after the introduction of the new policy<br>5. Follow up takes place after a sufficient period to allow the effects of policy change to become evident<br>6. Other factors that could have produced the change (other than policy) are identified<br>7. Criteria for evaluation are adequate and clear
Opportunities	Public opportunities:<br>1. Multiple stakeholders are involved<br>2. Primary concerns of stakeholders are recognized and acknowledged to obtain longer term support<br>Political Opportunities:<br>1. The political climate has either worsened or improved<br>2. Cooperation between public and private organizations has either worsened or improved<br>3. The lobby for the action has either worsened or improved
Obligations	1. The obligations of the various implementers are specified – who must do what?<br>2. Scientific results are compelling for action

Source: Cheung et al (2010) [30]
