# Supplementary material for: Policy analysis of the protection of Iranian households against catastrophic health expenditures: a qualitative analysis
Source: BMC Health Serv Res. 2023 May 5;23:445. doi: 10.1186/s12913-023-09275-0 (PMC10161991; doi:10.1186/s12913-023-09275-0)
Supplement: Supplementary file 4 — Additional file 4: Supplementary 4. Analysis of Iran stakeholders’ interest, knowledge level, power, and position. [file 12913_2023_9275_MOESM4_ESM.pdf]

Supplementary 4. Analysis of Iran stakeholders' interest, knowledge level, power, and position

Stakeholder	Stakeholder interest	Knowledge	Power/adjusted power	Position
Policy-making institutions (Iranian parliament, diagnostic council, etc.)	Decision maker	3	3.8	1.9
Governmental organization	Decision maker	3	4.0	1.7
Supreme Council of Insurance	Decision maker	5	3.7	2.1
Ministry of Health and Medical Education (MoHME)	Decision maker	5	4.0	1.7
Ministry of Cooperatives, Labor and Social Welfare (MoCLSW)	Decision maker	3	3.5	2.1
Service providers in the public sector	policy executive	4	3.3	2.3
Service providers in the private sector	policy executive	3	2.9	3.1
Health insurance organizations	High support for targeting	4	3.6	2.5
pressure groups	High support for targeting	2	3.0	2.9
mass media	Raising awareness	2	3.0	2.0
General population, charity organizations, and NGOs	Profit and raising awareness	2	2.7	2.0
International institutions	Policy maker	5	2.5	2.4

Stakeholders' knowledge, self-reported, and adjusted power was attributed on a five-point scale (1 = very low; 2 = low; 3 = medium; 4 = high; 5 = very high); stakeholders' position was attributed on a five-point scale (5 = high support; 4 = support; 3 = neural; 2 = opposition; 1 = high opposition).
